# Kalman Filter-Based Fusion of Collocated Acceleration, GNSS and Rotation Data for 6C Motion Tracking

**DOI:** 10.3390/s21041543

**Published:** 2021-02-23

**Authors:** Yara Rossi, Konstantinos Tatsis, Mudathir Awadaljeed, Konstantin Arbogast, Eleni Chatzi, Markus Rothacher, John Clinton

**Affiliations:** 1 Institute of Geodesy and Photogrammetry, ETH Zurich, Robert-Gnehm Weg 15, CH-8093 Zurich, Switzerland; awadaljm@student.ethz.ch (M.A.); karbogas@student.ethz.ch (K.A.); markus.rothacher@ethz.ch (M.R.); 2 Swiss Seismological Service, ETH Zurich, Sonneggstrasse 5, CH-8092 Zurich, Switzerland; jclinton@sed.ethz.ch; 3 Institute of Structural Engineering, ETH Zurich, Stefano-Franscini-Platz 5, CH-8093 Zurich, Switzerland; tatsis@ibk.baug.ethz.ch (K.T.); chatzi@ibk.baug.ethz.ch (E.C.)

**Keywords:** collocated vibration measurements, accelerometer, GNSS, rotational sensor, Kalman filter, data fusion, structural monitoring, wind turbine, seismology, robot

## Abstract

The ground motion of an earthquake or the ambient motion of a large engineered structure not only has translational motion, but it also includes rotation around all three axes. No current sensor can record all six components, while the fusion of individual instruments that could provide such recordings, such as accelerometers or Global Navigation Satellite System (GNSS) receivers, and rotational sensors, is non-trivial. We propose achieving such a fusion via a six-component (6C) Kalman filter (KF) that is suitable for structural monitoring applications, as well as earthquake monitoring. In order to develop and validate this methodology, we have set up an experimental case study, relying on the use of an industrial six-axis robot arm, on which the instruments are mounted. The robot simulates the structural motion resulting atop a wind-excited wind turbine tower. The quality of the 6C KF reconstruction is assessed by comparing the estimated response to the feedback system of the robot, which performed the experiments. The fusion of rotational information yields significant improvement for both the acceleration recordings but also the GNSS positions, as evidenced via the substantial reduction of the RMSE, expressed as the difference between the KF predictions and robot feedback. This work puts forth, for the first time, a KF-based fusion for all six motion components, validated against a high-precision ground truth measurement. The proposed filter formulation is able to exploit the strengths of each instrument and recover more precise motion estimates that can be exploited for multiple purposes.

## 1. Introduction

In recent years, Structural Health Monitoring (SHM) has gained significant ground due to its potential in automating structural assessment under operational, but also extreme event conditions (e.g., during earthquakes). Structural monitoring is most commonly achieved via use of vibration-based information, which is typically acquired via accelerometers (e.g., [1]), while more rarely Global Navigation Satellite System (GNSS) receivers are also adopted to further measure displacements [2,3]. However, these instruments only measure the 3 translational components and are missing the highly coupled rotational motion present as well. For example both, translational and rotational motions are relevant for the wind-excited motions of wind turbine structures, or even the ambient motions of an engineered structure, such as a building or a bridge. For SHM, rotations and translations offer important integral information, which are useful in correcting drifts that are typically associated with utilization of acceleration information alone, as is commonly the practice in vibration-based structural monitoring [3,4,5,6].

In a similar manner, ground motions that are triggered by an earthquake contain coupled translational and rotational components [7,8]. The six-component (6C) motion can include oscillating, as well as permanent motion [9]. An accelerometer will record any rotation around the horizontal axes as a translation, even though, in reality, it is simply gravitational leakage. Though the GNSS instrument is not susceptible to this effect, the GNSS antenna is influenced by rotation when positioned on a pole that is tilted during ground motion. The rotation around the z-axis could also induce a phase wind up error, although this would need a rotation of 18° to reach the noise amplitude of 1 cm. The instrumentation that is used to measure displacements, and subsequently earthquake magnitudes, can all be influenced by rotations.Therefore, it is important to measure the rotation if the true motion is to be understood [10].

We address this problem by adding an instrument to a proposed hybrid monitoring station for both structural and seismic monitoring, which can directly record these rotational components. Such an enhancement has been proposed in the domain of seismic monitoring by [7,9]. The use of rotational sensors for seismic applications has been applied for some years already [7,11,12,13,14,15,16,17,18,19,20,21]. Furthermore, we will combine the three datasets with a standard loosely coupled Kalman filter using co-located instrumentation. The filter allows for the coupling of the three measurements, despite the different sampling rates, noise levels, and frequency bands. This has already been successfully shown for the coupling of accelerometers with GNSS data, as described in [22]. In a number of applications, relating to seismic monitoring, this allows for a much more refined determination of the ground motions at a better resolution than using seismic-only solutions [23,24,25,26]. A 6C Kalman filter was later on shown by [19], where, for the first time, rotations were included in the filter procedure. In their filtering implementation the authors use the rotations to correct the noise-corrupt accelerometer measurements, but, in reality, the GNSS measurements are also affected by the rotations. Dependent on the length and offset of the antenna phase center to the point of rotation, the antenna will show rotation-induced translations. Because their study used the GNSS positions as the ground truth, they were not able to see the influence rotations have on them. By using the feedback system of our robot as the ground truth, our work shows that taking this parameter into account will improve the filter-extracted response estimation.

This study presents the results of fusing these three heterogeneous measurements via adoption of a linear Kalman filter (KF). In our KF setup, the GNSS data serve as the observation, whilst the pre-rotated acceleration and pre-rotated antenna phase center offset serve as inputs to the state and measurement equations of the filter. A detailed description is offered in Section 2.3. We use an experimental setup comprising an industrial six-axis robot arm in order to validate the results of the proposed fusion scheme. We use the robot as a motion generator and further use the feedback system as reference “ground truth”. The robot is exploited here as a first proof of concept for creating a controllable movement that can mimic structural response and allow for the verification and validation of the proposed algorithm. As the robot is unable to execute small amplitude and high-frequency motions that are typically linked to earthquakes or stiff structural systems, we opt for the simulation of an alternate type of structural response, namely the motion that results at the top of a wind turbine tower. Structural monitoring is particularly relevant for these types of structures, as their deformation is a decisive feature for their design [27], while their vibrational response carries important information on condition [28]. However, it is noted that the wind turbine motion is only used here as an example application, which is intended to illustrate the potential of the proposed 6C monitoring station for more general applications. During the performed robotic motion a GNSS antenna, an accelerometer and a rotational sensor are attached to a platform at the end of the arm (details are described in Section 2.1). A similar approach was followed in [29], where a 3C high-rate GNSS experiment was performed while using a similar robot system, which allowed for them to analyze the GNSS processing results for different baseline lengths between the moving and static antenna for relative positioning. Furthermore, Lin et al. [30] demonstrate a rotation correction scheme for accelerometer recordings, illustrated on motion generated by a robotic arm. In this work, we are, for the first time, demonstrating results from a 6C experiment, where a ground truth is available and used for a quality assessment of the instrument recordings. The proposed structural monitoring methodology is outlined in Section 2.1, with the verification of the filter results presented in Section 3.

## 2. Methodology

### 2.1. Experimental Setup

The proposed experimental setup for verifying a next generation monitoring station consists of three measuring instruments and a six-axis industrial robot arm, performing the 6C motions. The great advantage of using a robot arm lies in the fact that we have full control over the conducted experiment. While executing the prescribed motions, the robot features a feedback system for stabilizing its own trajectory. This comes with two main advantages. Firstly, it allows the robot to closely match the prescribed input motion. Secondly, it ensures the availability of a high-precision 6C ground truth, which serves for the validation of our instrumentation and the fusion process that is achieved by the suggested KF scheme. Nonetheless, minor stability issues may still occur at two locations: the connection to the concrete ground and the instrument platform itself. The robot is bolted to the ground via a 1.5 cm thick metal platform, which could be subject to minor bending. The bending of this platform would not be seen in the robot feedback, but recorded by the mounted instruments. Additionally, flexure of the steel platform on the robot arm, which is used to support the different instruments, could result in differential motion in the case of high frequencies, with respect to the motion executed by the robot. Both of these occurrences are considered to be negligible in our experiment, since the motion is well within the robot limitations, and the platform is rigid.

Although the motion of the robot is highly repeatable, three main limitations arise: (1) there is a weight limit of 7 kg that we can load onto the robot arm, which restricts the instruments loaded for a single experiment—this is especially important for the rotational sensors. Instruments that are typical in geodesy and classical seismology include high-quality instrumentation with low weight, but there is currently no rotational sensor with similar quality-to-size proportions [31]; (2) during large accelerations, the axes of the robot arm are subject to large forces, which will initiate a full stop if they overstep a certain threshold; and, (3) the smallest rotation steps are at 0.005°, which limits the sensitivity with which we can perform earthquake-like motions.

**Figure 1:**
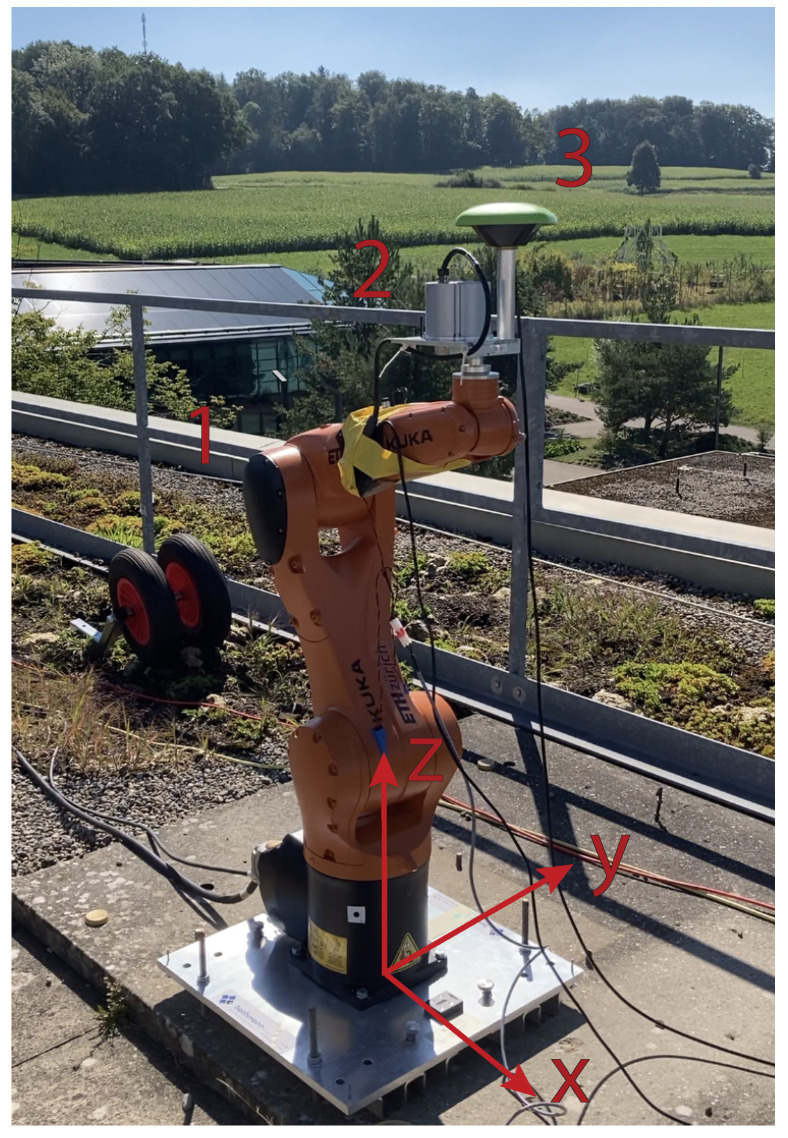
The experiment set-up showing (1) the KR AGILUS-2, KR 6 R900 sixx robot arm, (2) KVH 1750 IMU, and (3) Javad GrAnt G3T Global Navigation Satellite System (GNSS) Antenna. The location of (2) is also where the EpiSensor is placed.

Given the robot’s limitation in generating low amplitude rotations, experiments of rather large amplitudes need to be carried out, allowing for use of a light-weight, but high-quality, inertial measurement unit (KVH 1750 IMU). While this suffices for these proof-of-concept experiments, this device is not ideal for general monitoring, as its response is not sufficiently broad-band to capture strong earthquake motions, and it does not have the resolution to capture low amplitude motions on typical infrastructure. Additionally, we used a Javad antenna and Septentrio receiver, as well as an EpiSensor and Centaur digitizer to measure the performed motions in displacement and acceleration. The specifications of all three instruments are listed in Table A1. To have both the accelerometer and the rotational sensor placed near the point of rotation, we mounted these separately and carried out the experiment twice. The instrument setup, together with the orientation of the three axes of the local coordinate frame, can be seen in Figure 1, below.

**Figure 2:**
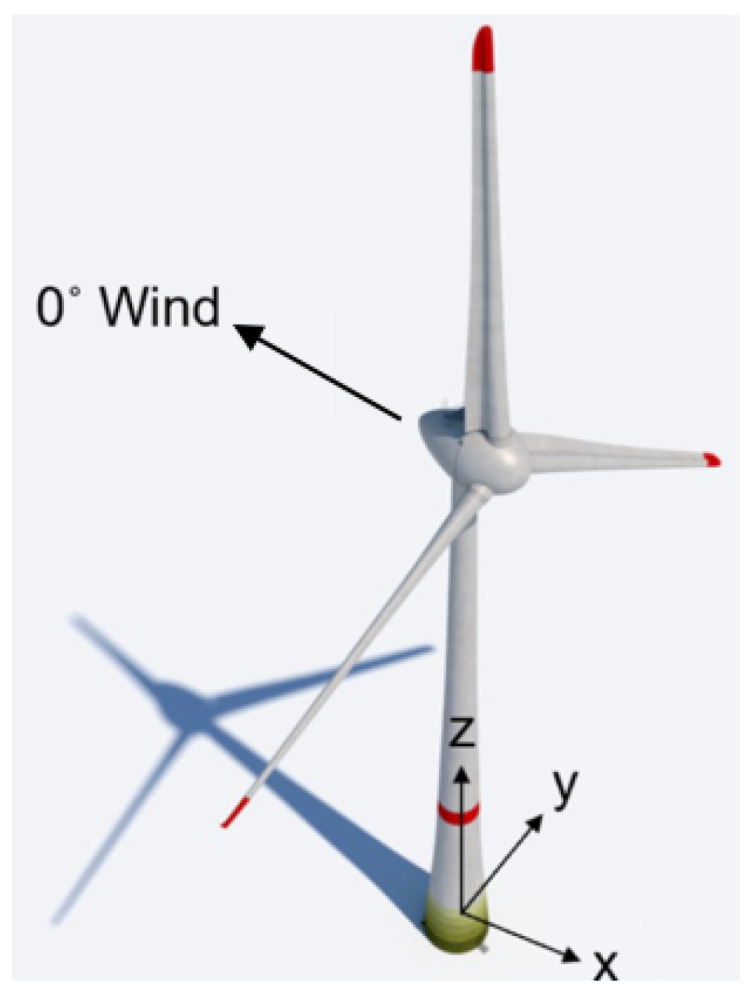
The experiment that was conducted on the robot simulates the motion at the top of a wind turbine excited by wind. The amplitudes have been adapted to show a diverse range of motion.

The motion performed in the experiment is 300 s in duration and includes translations in the horizontal axes as well as rotations around them. No motion is executed along or about the z-axis. Nonetheless, low-amplitude and high-frequency ticks are visible in the vertical axis, due to changes of the rotation direction of the robot axis. The basis for our experiment is the motion on top of a wind turbine tower, during wind excitation, as seen in Figure 2. The simplified motion includes five main frequencies, where the translation in the *x*-axis was coupled with the rotation around y and vice versa. The dominant frequency range is 3 Hz. Table A2 shows the exact values and amplitudes. We performed three different amplitude sets for these frequencies and performed them twice. Table 1 shows the different amplitudes for the three experimental sets. The naming is designed to facilitate comprehension for the reader. T: translation, R: rotation, L: large, S: small.

**Figure 3:**
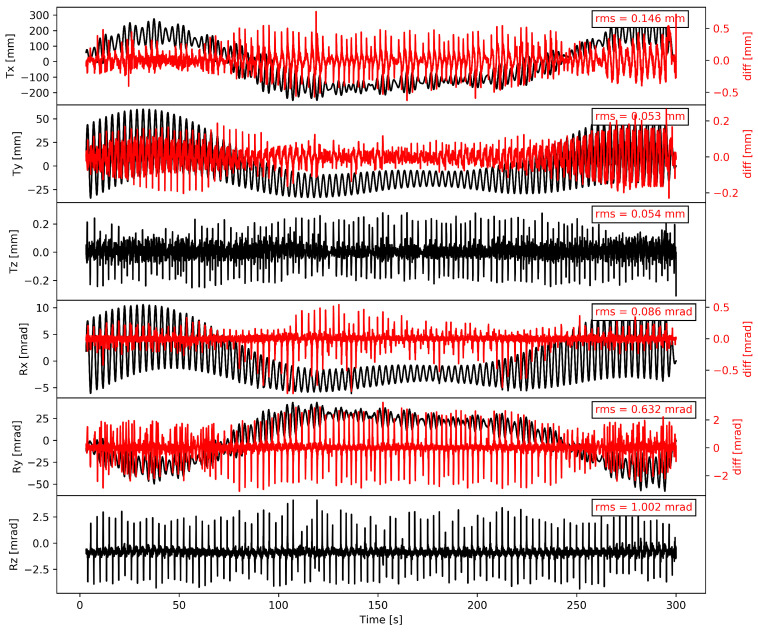
Time series of the robot feedback (RFB) of experiment TLRL as displacements and rotations in and around all three axes in a right-hand coordinate system in black. The z-axis input was set to zero for translations and rotations, but the robot requires axis direction changes when performing the experiment and these are seen as high-frequency ‘ticks’. These high-frequency offsets or ‘ticks’ are observed in the measured RFB signal, a feature of the robot arm. In red, the difference of the RFB to the programmed input motion is visible with the corresponding root mean squared error (RMSE) that is noted in the upper right corner.

Set 1 (TLRL) is characterized by large rotations and large translations. Set 2 (TLRS) has the same translation amplitude but lower rotations. Set 3 (TSRS) uses small amplitudes in both, translations and rotations. Figure 3 depicts the experiment TLRL, including the difference of the robot feedback (RFB) to the input motion and the root mean squared error (RMSE) thereof. The three different experimental sets will demonstrate the following five challenges: low amplitudes for GNSS as compared to the noise level, GNSS antenna phase center corrections, large gravitational leakage for the accelerometer, low-frequency motion for the accelerometer, and low-frequency motion for the rotational sensor. These instrumentation challenges, as well as the Kalman filter performance, will be analyzed in comparison to the robot feedback.

Because we are using the robot feedback as a ground truth for the accomplished motion and, therefore, as a reference for comparing the KF estimates, it is important to determine the quality and repeatability of these signals. This was analyzed by comparing the four repetitions of one experiment with each other and calculating the RMS of the discrepancies (error) in terms of the translations and rotations. The RMSE is calculated by using the difference of all experiment pairs ($exp_{j}$ and $exp_{i}$), resulting in six RMSE values, as seen in the following equation:

(1) $$RMSE_{mean}=\frac{1}{6}\sum_{i=1}^{3}\sum_{j=i+1}^{4}\sqrt{\sum_{n=0}^{N}\;\frac{{\left(exp_{j,n}-exp_{i,n}\right)}^{2}}{N}},\;for\;N=length\;of\;signal$$

**Figure A1:**
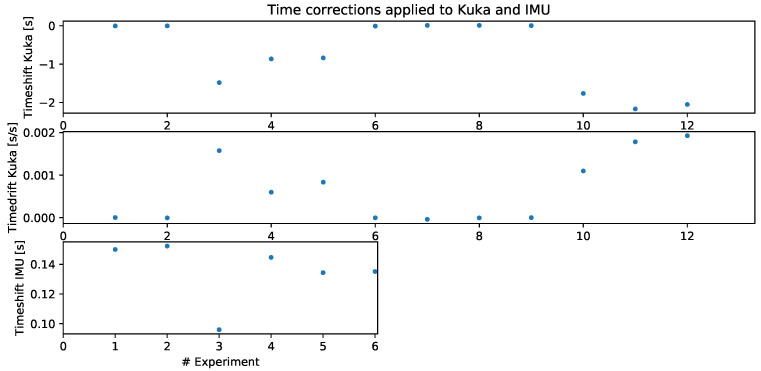
The figure shows the time shift and drift applied to the RFB and IMU data sets. The experiments 3,4,5,10,11,12 do not have linear time shifts, therefore the corrections still do not allow a good comparison. TLRL: 1,2,7,8; TLRS: 3,4,9,10; TSRS: 5,6,11,12.

Table 2 shows the mean of the resulting six RMSE error values per experiment and per axis. The TLRL and TSRS experiments show very low RMSE values of below 0.35 mm in the three translational axes. The RMSE discrepancy in the rotational axes appear lower for large motions with values between 0.16 and 0.02 mrad than for lower amplitude motions with 0.28 and 0.07 mrad. This is expected, since the robot is at its resolution limit for small rotations. The TLRS experiment yields worse results than the other two. With a translational RMSE discrepancy five to 30 times larger than for the other experiments, the quality is definitely lower. The reason for the high RMSE is primarily attributed to the lack of precise synchronization, as described in Figure A1 in the Appendix. The sub-mm RMSE values of the RFB during experiments, for which a better synchronization is achieved, allow for us to compare the instrument recordings of the different repetitions. Therefore, we can trust that the robot has high-quality repeatability, allowing for us to combine instrument recordings that were collected over different experiments, as well as use the RFB from different repetitions to compare against each other. For this reason, we use the one repetition of the TLRS experiment that features precise synchronization as the ground truth for all TLRS experiments. We are aware that not mounting the instruments simultaneously will induce additional errors deriving from the noise from different repetitions. However, it was the only way to ensure that the IMU as well as the EpiSensor can be as near as possible to the point of rotation of the robot arm. Otherwise, the EpiSensor would have been subject to additional and larger effects that originate from apparent forces (Centrifugal, Coriolis, and Euler forces), since all three are dependent on the length of the offset between point of rotation and the instrument explained in Section 3.1.

### 2.2. Pre-Processing

Perhaps a main hurdle in merging different instruments into a combined motion solution lies in time synchronization. The design of a robust monitoring station requires a single timing device, which provides the timing for all deployed instruments. This assures synchronized time stamps and, therefore, better quality of the sensor fusion. Such a timing device was not yet implemented in our study, so time corrections had to be applied in emulating the final solution see Table A3. The procedure for time correction is described in more detail in Appendix A.3. Additionally, we independently and separately mounted the accelerometer or the IMU and GNSS antenna as near as possible to the rotation point and performed the three experimental sets twice in order to have both the accelerometer and IMU close to the point of rotation. This means that, in reality, 12 individual experiments were setup, with the EpiSensor (accelerometer) data shifted in time to match the IMU recordings, resulting in six experiments to be fused in the KF.

The GNSS raw data are processed with the commercial GrafNav software in relative positioning mode, using a very short baseline of 5 m. We are using GPS, GLONASS, Galileo, and Beidou satellites for our L1/L2 solution with a sampling rate of 100 Hz. An L1/L2 solution means that we use the signals of the two frequencies L1 and L2 for GPS/GLONASS, E1 and E5b for Galileo, and B1l and B2l for Beidou to determine the positions. This is the best possible solution for GNSS, yielding the lowest noise. The observation time series for the filter only includes the horizontal components. The vertical (z) component is assumed to equal zero, since the high-frequency tick motion of the robot that is seen in Figure 3 is well below the noise level of the GNSS.

All of the instrument recordings, as well as the robot feedback (RFB), are interpolated to the EpiSensor time-stamps with a sampling rate of 250 Hz for the IMU, EpiSensor, and RFB data, and a sampling rate of 100 Hz for the GNSS data. The IMU data are integrated and high-pass filtered with a corner frequency of 0.001 Hz, while the other instruments are left unfiltered.

### 2.3. 6C Kalman Filter

The methodology that is chosen for the combination of the three instruments is the standard sensor fusion linear Kalman filter (KF). An advantage is the ability of combining diverse instruments, measuring different quantities and at different sampling rates. These measurements are then assigned distinct weights through appropriately configured process and measurement noise co-variance matrices, which optimize the combined input to output the desired estimated quantities. In the field of navigation and odometry, the KF is a renowned methodology, which has been in use for decades [32]. The KF combination of GNSS with an accelerometer sensor for seismic applications has first been successfully shown by [23]. Almost a decade later, the rotations were included for the first time into the filter equations to correct the orientation of the acceleration sensor [19]. The basis of the proposed configuration is taken from [23] and it is developed further for use with the 6C configuration. Two pre-computations are executed prior to the filter itself, namely the integration of the angular velocity ${\dot{r}}_{k}$, see Equation (2), and the rotation of the acceleration recordings $a_{k}$ into the local coordinate system, see Equation (4). The filtering and integration could be implemented within the filter itself, allowing for real-time application. The goal of this paper was not a real-time implementation, so it is not done here. The angular velocities are integrated with a simple trapezoidal rule for time steps k−1 and *k* that are separated by dt, as in Equation (2). The resulting angles $r_{k}$ are then input into the Euler rotation matrix $R_{k}$ in Equation (3), which is used to rotate the acceleration recordings back into the local frame in Equation (4). GNSS antennas are often attached to a pole with varying length and the tilting of this pole can potentially introduce additional translations. In our case, the short antenna pole is also offset in a positive y-direction, since the IMU and EpiSensor are situated over the point of rotation, as seen in Figure 1. Coupled with the antenna phase center offset, the vector from the point of rotation to the antenna phase center is $h=\left[\begin{array}{c} -7.76\;\mathrm{mm}\;51.69\;\mathrm{mm}\;213.28\;\mathrm{mm} \end{array}\right]$. With the knowledge of the occurring rotations, we calculate the displacements that arise with Equation (5), and use them during the filter process.

(2) $${\dot{r}}_{k}=\left[\begin{array}{c} {\dot{\phi }}_{k}\\ {\dot{\theta }}_{k}\\ {\dot{\psi }}_{k} \end{array}\right],\;\;\;\;\;\;r_{k}=\left[\begin{array}{c} \phi_{k}\\ \theta_{k}\\ \psi_{k} \end{array}\right]=r_{k-1}+\frac{{\dot{r}}_{k-1}+{\dot{r}}_{k}}{2}\;dt$$

(3) $$R_{k}=\left[\begin{array}{ccc} c_{\theta }c_{\psi } & c_{\theta }s_{\psi } & -s_{\theta }\\ s_{\phi }s_{\theta }c_{\psi }-c_{\phi }s_{\psi } & s_{\phi }s_{\theta }s_{\psi }+c_{\phi }c_{\psi } & c_{\theta }s_{\phi }\\ c_{\phi }s_{\theta }c_{\psi }+s_{\phi }s_{\psi } & c_{\phi }s_{\theta }s_{\psi }+s_{\phi }c_{\psi } & c_{\theta }c_{\phi } \end{array}\right],\;\;\;\;\;\;with\;\;c_{\theta },s_{\theta }:cos\left(\theta_{k}\right),sin\left(\theta_{k}\right)$$

(4) $$a_{k}^{r}=\left(R_{k}\cdot \left(a_{k}-g\right)\right)+g,\;\;\;\;\;\;with\;\;g:gravitational\;\;acceleration$$

(5) $$h_{k}^{r}=R_{k}\cdot h$$

The state (process) equation of the KF is designed while using the standard adoption of displacement and velocity in the state vector x. The measured and pre-rotated acceleration $a^{r}$ and the rotated height vector $h^{r}$, serve for formulating the KF input vector u, as defined in Equation (6).

(6) $$x_{k}={\left[\begin{array}{c} d_{x}\\ d_{y}\\ d_{z}\\ v_{x}\\ v_{y}\\ v_{z} \end{array}\right]}_{k}=\underset{A}{\underset{︸}{\left[\begin{array}{cccccc} 1 & 0 & 0 & dt & 0 & 0\\ 0 & 1 & 0 & 0 & dt & 0\\ 0 & 0 & 1 & 0 & 0 & dt\\ \; & \; & & 1 & 0 & 0\\ \; & 0_{3\times 3} & \; & 0 & 1 & 0\\ \; & \; & & 0 & 0 & 1 \end{array}\right]}}x_{k-1}+\underset{B}{\underset{︸}{\left[\begin{array}{cccccc} \frac{dt^{2}}{2} & 0 & 0 & \; & & \\ 0 & \frac{dt^{2}}{2} & 0 & \; & 0_{3\times 3} & \\ 0 & 0 & \frac{dt^{2}}{2} & \; & & \\ dt & 0 & 0 & \; & & \\ 0 & dt & 0 & \; & 0_{3\times 3} & \\ 0 & 0 & dt & \; & \end{array}\right]}}u_{k}+w_{k}$$

where $u_{k}={\left[\begin{array}{cccccc} a_{x}^{r} & a_{y}^{r} & a_{z}^{r} & h_{x}^{r} & h_{y}^{r} & h_{z}^{r} \end{array}\right]}_{k}^{T}$, w is the process noise vector, which is assumed to be drawn from a zero mean multivariate normal distribution with covariance Q. A and B are the state and input matrices.

The only observations, which are included in the observation vector $y_{k}$ of the devised KF, pertain to the GNSS positions $p={\left[p_{x}\;p_{y}\;p_{z}\right]}^{T}$, as seen in Equation (7).

(7) $$y_{k}={\left[\begin{array}{c} p_{x}\\ p_{y}\\ p_{z} \end{array}\right]}_{k}=\underset{C}{\underset{︸}{\left[\begin{array}{ccc} 1 & 0 & 0\\ 0 & 1 & 0\\ 0 & 0 & 1\\ & \; & \\ & 0_{3\times 3} & \\ & \; \end{array}\right]}}x_{k}+\underset{D}{\underset{︸}{\left[\begin{array}{ccc} & \; & \\ & 0_{3\times 3} & \\ & \; & \\ 1 & 0 & 0\\ 0 & 1 & 0\\ 0 & 0 & 1 \end{array}\right]}}u_{k}+z_{k}$$

where z is the process noise vector, which is assumed to be drawn from a zero mean multivariate normal distribution with covariance G. C and D are the feedthrough (or feedforward) matrices, respectively.

Table 3 summarizes the steps of the linear KF. The prediction step utilizes the state equation of the filter to obtain a prior estimate of the state, $x_{k|k-1}$. Here, the notation $x_{i|j}$ is used to denote the estimate of the state x at time step *i*, being conditioned on information up to time step *j*. The prior estimate of the covariance matrix $P_{k|k-1}$ of the state vector is estimated using the information from time step k−1, as well as the process noise variance matrix Q. The availability of measurements is not yet taken into account. In the subsequent update step, the innovation ${\mathrm{dy}}_{k}$, i.e., the difference between the actually measured GNSS data, $y_{k}^{\mathrm{GNSS}}$, and the KF-predicted prior estimate of the displacement $y_{k|k-1}$ is calculated as ${\mathrm{dy}}_{k}=y_{k}^{\mathrm{GNSS}}-C\;x_{k|k-1}-D\;u_{k}$. This is later used for the correction of the prior given the measurement information at time step *k*, i.e., the posterior KF estimate $x_{k|k}$. For this, the Kalman gain K needs to be further calculated, through the variance of the state vector and the measurement noise variance matrix G. The Kalman gain is finally used as a weight (gain) to correct the prior estimate of the state variance $P_{k|k}$, as well as the state itself, $x_{k|k}$, as outlined in the summary equations of Table 3.

At this point, it is recalled that the measurements that are to be fused are acquired at different sampling rates. More specifically, the GNSS unit employs a 100 Hz sampling rate, while the IMU and rotational sensor sample at 250 Hz. The KF naturally allows such a multi-rate sensor fusion. The KF states are estimated at 250 Hz, which requires a linear interpolation at every second GNSS 100 Hz time step to offer observations that align with integer multiples of the 1/250 time step of the process equation of the KF. For the time steps, where no GNSS observations are available, the filter operates in prediction mode, while using the process equation, and the update step is skipped. Thus, the prior predicted state and the state variance are not corrected with the Kalman gain, but are directly taken as the posterior. The GNSS sampling rate is high enough to ensure that the drifts that are caused by numerical integration (process equation) are minimal.

The process and measurement noise covariance matrices Q and G allow for tuning the filter, based on the confidence that is attributed to the state equations (integration) and the assumed reference observations (GNSS). The tuning refers to the amount of correction that is applied to the state vector through the Kalman gain in the update step of Table 3. These covariance matrices can be auto-tuned within the filter itself, by quantifying the actual model errors [33]. However, for this study, we are keeping these matrices constant (time-invariant) during the filter procedure. The chosen values are based on the RMSE of the difference between the instruments and robot feedback (RFB), and they have additionally been tuned based on how well the KF estimation agrees with the RFB. In Table 4, the values for Q and G are explicitly shown. The TSRS experiment features slightly different Q values, because the translation amplitudes are lower for this experiment.

In order to assess the influence the rotations of the two instruments, the accelerometer and the GNSS antenna, have on the KF combination, we perform three different realisations; (*I*) full KF combination, including the effect of rotation on both, the GNSS and EpiSensor, (*II*) only taking account of rotations on the EpiSensor, ignoring the effect of rotation on the GNSS and (*III*) excluding rotation completely. In comparison to (*I*), we do not correct for the rotation of the antenna pole in (*II*) and Equation (5) becomes $h_{k}^{r}=h$. In (*III*), we set $r_{k}$ to zero, which, in practice, results in Equations (4) and (5), becoming $a_{k}^{r}=a_{k}$ and $h_{k}^{r}=h$.

## 3. Results

The proposed Kalman filter aims at reproducing the true 6C motion performed by the robotic arm. By including the influence of the rotations in the input vector, we obtain rotation-free state estimates. While the setup of the filter equations already gives a direct weight to each system, the covariance matrices additionally allow for experiment-specific tuning. The measured quantity that was employed as KF observation $y_{k}$ is assumed to be the most trustworthy; here, GNSS position recordings. This section elaborates on the influence of the equation setup and the tuning of the process and measurement noise covariance matrices on the prediction results.

### 3.1. Influence of Rotations on Acceleration

**Figure 4:**
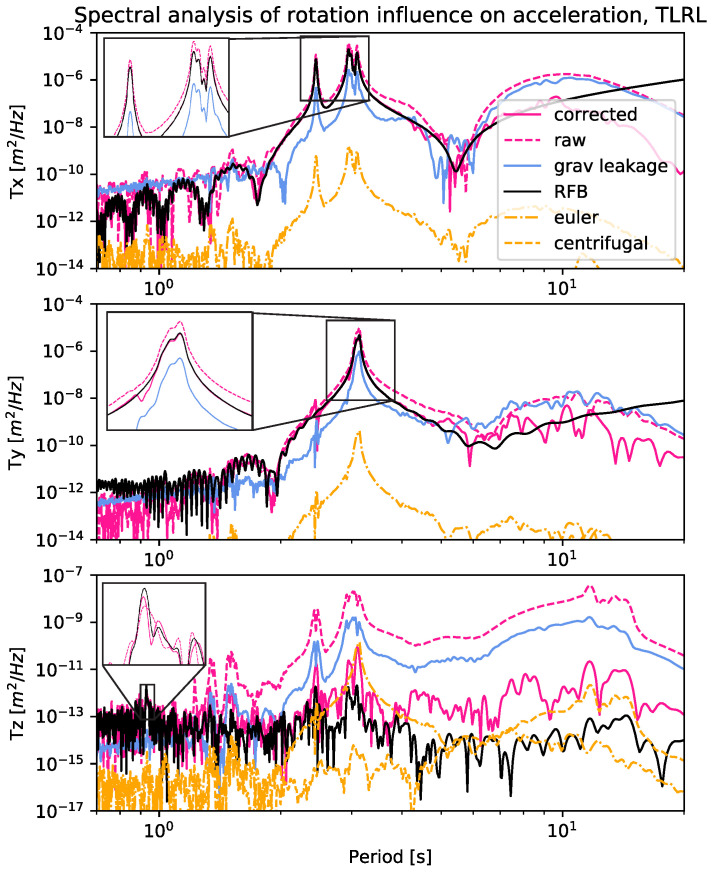
The figure includes six datasets; ‘raw’ is the EpiSensor data, ’corrected’ denotes the back rotated acceleration, ‘grav leakage’ is the rotated gravitational vector, ’RFB’ is the robot feedback and the two calculated apparent forces converted to displacements are denoted in yellow. The insets zoom into the key frequency peaks per axis.

For an inertial sensor, such as the accelerometer, rotations about a horizontal axis lead to a leakage of the gravitational acceleration into the horizontal axes and out of the vertical axis, producing spurious horizontal and vertical translations. It is not only the gravitational vector that affects the measurement, but also the misorientation of the sensor. Additionally, the apparent forces, such as Centrifugal, Coriolis, and Euler components, induce additional accelerations that can lead to a misinterpretation of the data. Equations (8)–(10) denote how the calculations are performed, with $\dot{r}$ the angular velocity of the robot and with $b_{inst}=\left[0\;\mathrm{mm}\;\;30\;\mathrm{mm}\;\;50\;\mathrm{mm}\right]$ designating the distance from the point of rotation to the EpiSensor instrument. In the absence of precise knowledge of where each force feedback module is situated inside the instrument case, the calculation of each of these three acceleration components cannot be precise. Because $b_{inst}$ is constant, no Coriolis force is induced by the robot angular velocity. Therefore, we have calculated the power spectrum of the other two apparent forces and plotted it together with the RFB data to obtain an estimate of the possible amplitude. Figure 4 shows that with the assumed offset $b_{inst}$ from the point of rotation, the apparent forces do not have a significant impact on the accelerometer data. In view of the minor effects, we have decided to omit a correction procedure for these terms.

(8) $$a_{centrifugal}=\dot{r}\times \left(\dot{r}\times b_{inst}\right)$$

(9) $$a_{coriolis}=2\left(\dot{r}\times {\dot{b}}_{inst}\right)$$

(10) $$a_{euler}=\ddot{r}\times b_{inst}$$

Figure 4 illustrates how the raw acceleration overshoots at the main periods of motion around 3 s in the horizontal axes x and y. At these same periods, the gravitational leakage is of course significant. The correction for the gravitational leakage then results in the corrected data set, which nicely follows the black RFB line until around a period of 10 s. Beyond 10 s the accelerometer begins to severely deviate. This is mostly due to the fact that long-period motions have very small accelerations that are close to or below the resolution of the sensor, so sensor noise is dominant following the double integration procedure. In the vertical component the largest motion of the robot is attributed to the high-frequency ticks, originating from a change in the rotation direction of one of the axes of the robot arm. These high-frequency ticks are seen in the zoom of the Tz subfigure.

### 3.2. Influence of Rotations on GNSS Displacement

**Figure 5:**
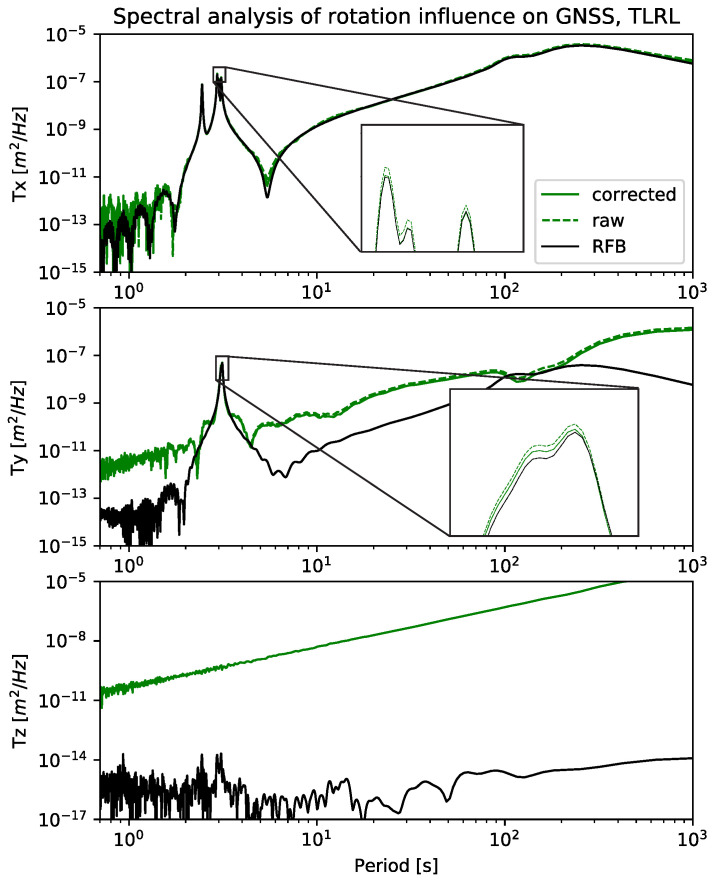
The figure includes three data sets; ‘raw’ is the measured GNSS data in displacement, ‘corrected’ denotes the back rotated GNSS data in displacement; and, ‘RFB’ is the robot feedback. The zoomed-in parts are only shown for the horizontal axes, because the vertical axis of GNSS only shows noise.

The influence of rotations on the GNSS data is not only dependent on the length and offset of the antenna, but also on the distance to the antenna phase center from the point of rotation. In this case study, the point of rotation is very well known and, therefore, the vector from the point of rotation to the antenna phase center can be easily and precisely determined. The vector is, in our case, h=[−7.76mm51.69mm213.28mm]. Even though it is actually quite short, the distortion that is caused in the GNSS data is visible and has to be corrected. Figure 5 shows the spectral power of the GNSS positions without any processing, as compared to the rotation-corrected GNSS positions and the RFB. The zoomed-in subfigure shows that the horizontal amplitudes are diminished and fit better, although still not perfect, to the RFB. It has not been possible to determine why the GNSS cannot be fully corrected. This may be due to further factors, apart from the antenna phase center offset, which are influenced by the rotations, or perhaps a further source, beyond the rotations, is distorting the data. For motions with a frequency higher than 1 Hz, the setting of the bandwidth of the loop filter can have a serious impact [34] and distort either the amplitude or induce a phase error. In our case, the frequency is lower than 1 Hz, hence the bandwidth of the loop filter should not influence it. The noise amplitude on the z-axis is very high and, therefore, the rotation correction is negligible.

### 3.3. Kalman Filter Measurement Fusion

Each instrument that is involved in the fusion process has different strengths and weaknesses. The high noise level of 5 mm in the GNSS vertical positions and the low sampling rate, leads to extremely poor quality in resolving Tz. The EpiSensor, on the other hand, has a higher sampling rate and a multiple orders lower noise level and can, therefore, resolve Tz much better. However, the EpiSensor measures acceleration, which is not stable after a double integration without filtering, while the GNSS antenna directly measures displacement and can, therefore, resolve Tx and Ty quite well. Both of the instruments are influenced by rotations, the EpiSensor more than the GNSS antenna, which we correct by using the data from the rotational sensor in the IMU. The root mean square error (RMSE) of the difference between the instruments and the robot feedback (RFB) in comparison to the RMSE of the difference between the KF results and the RFB indicates that the fusion results in the combination of the individual strengths.

**Figure 6:**
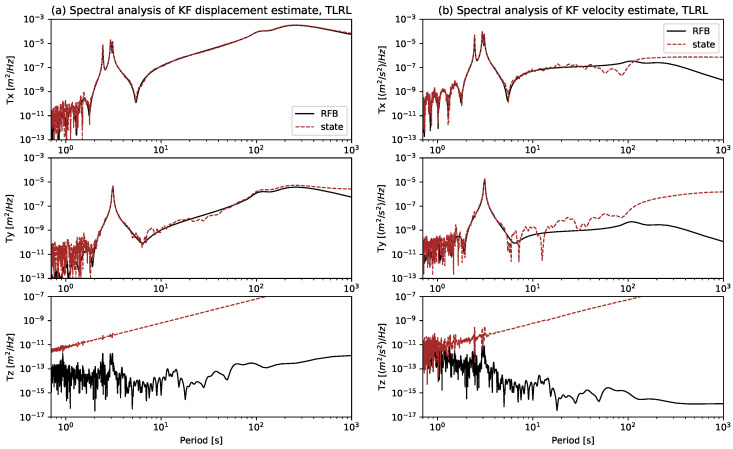
The robot feedback (RFB) is plotted in black and the KF estimate (state) is plotted in brown for the TLRL experiment. The (**a**) shows the KF displacement estimate and (**b**) shows the KF velocity estimate.

Figure 6a,b show the spectral analysis of the KF displacement and velocity estimates for the TLRL experiment, and they demonstrate the good accordance of the KF estimates in brown with the RFB in black during the main periods. Not only the short periods around 3 s fit satisfactorily, but also the long periods on the horizontal axes are resolved quite well. Appendix A.4 shows the spectral analysis of the TLRS and TSRS experiments.

**Figure 7:**
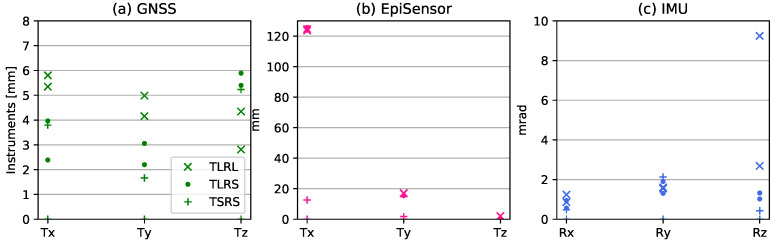
The RMSE values in comparison to the RFB for both repetitions of the three experiments in Tx, Ty, Tz. The GNSS positions plotted (**a**) are not corrected for the antenna offset rotation. The accelerometer data (**b**) is integrated twice, high-pass filtered at 0.09 Hz and not corrected for rotation of the instrument. The angular rate data (**c**) was integrated and high-pass filtered at 0.001 Hz. The subplots have different scales on the *x*-axis for better visibility.

**Figure 8:**
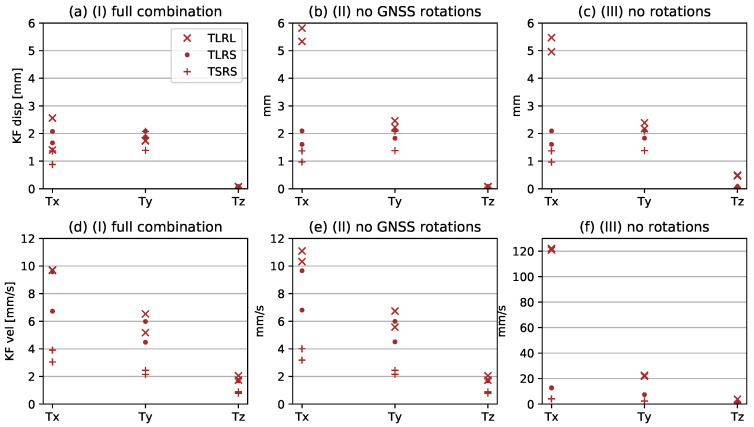
Summary of KF performance in terms of RMSE. (**a**–**f**) show the KF estimates of displacement and velocity. The (**a**) and (**d**) show the RMSE when applying the (*I*) full combination method. (**b**) and (**e**) ignore the rotation of GNSS (*II*). (**c**) and (**f**) assume that no rotational information is available (*III*). The subplots have different scales on the *x*-axis for better visibility.

**Figure 9:**
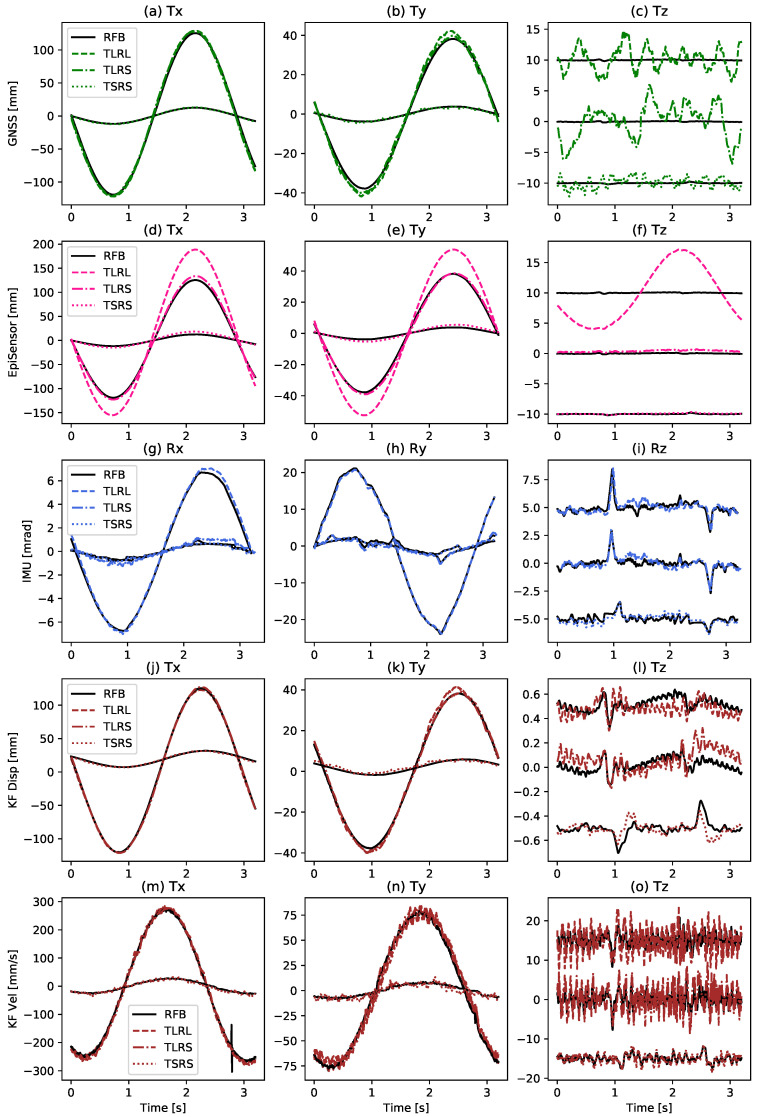
A summary of instrument (top three) and KF (bottom two) performance using time series. Each row of subfigures shows a 3 s zoom-in at 300 s of the simulated wind turbine tower with the recorded robot feedback (RFB) in black and additionally an instrument (**a**–**i**) or the KF estimated quantity (**j**–**o**) in color. All three experiments TLRL, TLRS and TSRS are shown per subfigure. (**a**–**c**) shows the GNSS data, (**d**–**f**) the EpiSensor data, (**g**–**i**) the IMU data, (**j**–**l**) the KF displacement estimates, and (**m**–**o**) the KF velocity estimates. The data have been high-pass filtered with a corner frequency of 0.08 Hz. Tz of each experiment was manually shifted for better visibility.

Figure 7 presents an overview of the performance of the individual sensors and Figure 8 presents the different realisations of the KF; (*I*) full KF combination, including the effect of rotation on both GNSS and EpiSensor, (*II*) only taking account of rotations on the EpiSensor, ignoring the effect of rotation on the GNSS and (*III*) excluding rotation completely. The RMSE of the difference of each instrument to the robot feedback (RFB) and the difference of the KF estimates to the RFB is shown for both repetitions of all three experiments: TLRL, TLRS, and TSRS. The errors of the GNSS positions are within the expected values of 1–8 mm, for the high-rate ultra-short baseline solution. The plotted GNSS data are not corrected for the rotations occurring, which is visible in the higher horizontal RMSE of the TLRL experiment, as opposed to the TLRS, which exhibits lower rotations. The RMSE values for Tz are only an indication of the noise level on the vertical axis, since the actual motion of the robot on Tz is far beneath this noise level. While the GNSS data are unfiltered, the accelerometer data are high-pass filtered at 0.09 Hz to calculate the RMSE, while it is actually used unfiltered within the KF fusion scheme. Without this filtering procedure, the double integration would result in an exponential drift. Unfortunately, this filter also results in high RMSE values for Tx. The long period motion performed by the robot is filtered out of the acceleration data and results in the 125 mm RMSE. The RMSE of the acceleration in Tz, on the other hand, yields extremely low values for all of the conducted experiments. The IMU, as plotted in Figure 7c, has an RMSE lower than 2.1 mm overall, with the exception of Rz of the TLRL experiment, where the large rotations are performed. Additionally, the Ry axis has slightly worse RMSE than the Rx, which can be explained by the unsmoothened robot performance. During the small rotations, the robot is at its limit and cannot always execute these smoothly, as seen in Figure 9h.

Figure 8a–c show the RMSE between the reference displacement RFB and the Kalman filter estimate of the displacement state, while Figure 8d–f show the corresponding RMSE of the velocities. The TLRL displacements exhibit much higher RMSE values, as shown by the comparison Figure 8a,b, which are caused by the rotation of the vector between the antenna phase center and the point of rotation. By taking into account that the antenna phase center is influenced by rotations, we are able to halve the RMSE, thus refining the estimated motion result. For small rotations, as are present for the experiments with RS in the name, this effect is negligible, as shown by comparing the TLRS and TSRS experiments in Figure 8a,b. If we choose to not correct the accelerations for the effect of rotations, only minor influence is noted on the horizontal axes. It is only the vertical component Tz that shows significant diminishing in estimation quality when the correction for rotationally induced gravitational influences is omitted. This shows that our filter heavily relies on the GNSS measurement for ensuring precision of the horizontal component estimates, but it relies much more on the acceleration input for the vertical (z-component) estimation. This is achieved by setting the z-component of the GNSS positions to zero. Similar to a high-pass filter, this stabilizes the double integration and alleviates drifting, without actually having to apply a filter to the acceleration records.

In contrast, the accelerometer data are essential in the precise estimation of the velocities. Ignoring rotation correction for the GNSS positions that are seen in Figure 8e in comparison to Figure 8d only slightly worsens the Tx and Ty RMSE for the TLRL experiment, while not affecting the other two experiments at all. The large influence of rotations on the accelerometer data is seen in Figure 8f, where both, the accelerometer and the GNSS data are not corrected for the occurring rotations. Tx for the TLRL experiment exhibits a more than ten times larger RMSE in Figure 8f as compared against Figure 8e, where the accelerometer data are corrected for rotationally induced influences, but the GNSS is not. Even for the case of small rotations, as in TLRS, an impact is noted on the estimation results in Figure 8f, albeit smaller.

While the RMSE values offer a good overview of the instruments’ performance, the time domain representation of the signals can offer a better indication of the weaknesses of each instrument, as well as help appraise the precision of the KF estimation. A three second zoom-in at 300 s of the simulated wind turbine tower with the recorded RFB signal is presented in Figure 9, which shows the largest amplitude of the signal. For better comparison, the signals of all three experiments are shown in the same subfigure and the horizontal axes are demeaned to zero-offset, while the vertical axis is shown with an offset. Additionally, all three instruments (top) and the estimates of the full Kalman filter fusion (bottom) are plotted. The GNSS horizontal positions show a good correlation to the true displacements with only a small overshoot, since they are only slightly affected by rotations. The TLRL sine wave overshoots by ca. 2.7% and the TLRS overshoots by ca. 1.7%. The z-component, on the other hand, only shows noise and it is not able to record any motion from the robot. For the EpiSensor data (Figure 9d–f), it is clear that the TLRL displacement strongly overshoots the true amplitude seen as the solid black line by around 50%. Additionally, for the small rotations an overshoot of ca. 8% is observable for the large translations (TLRS) and ca. 50% for the small translations (TSRS). The IMU (Figure 9g–i) only shows minor inconsistencies, which are most likely due to the reduced quality of the rotational sensor.

Figure 9j–l indicate the KF displacement estimates. The sine waves are very smooth in Tx and in Ty, but, nonetheless, coincide well with the RFB. The large overshoot of the EpiSensor and the minor overshoot of the GNSS positions have been filtered out, leaving a relative accurate representation of the RFB. Some GNSS noise is still present and we have not been able to completely correct for it. Figure 9l shows the vertical component, where Tz follows the RFB, primarily following on the EpiSensor data. A comparison of the state velocities to the RFB in Figure 9m–o, proves that the KF estimates are able to reproduce the correct amplitudes and frequencies. Nonetheless, the KF velocity estimates are noisy in comparison to the displacements.

**Figure 10:**
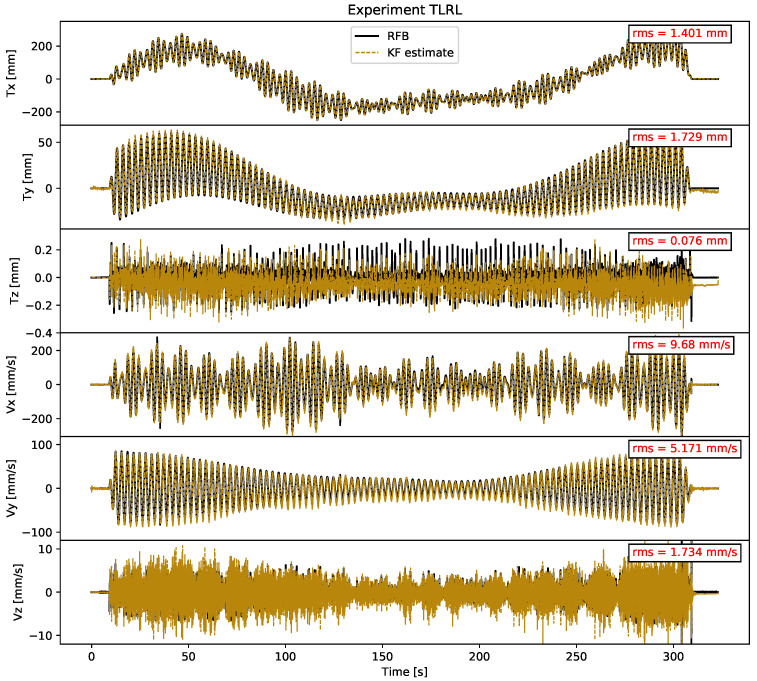
The figure illustrates the time series of the KF estimates for the TLRL experiment in yellow plotted against the robot feedback (RFB) in black. The top three subfigures show the KF displacement estimates versus the RFB. The lower three subfigures show the KF estimated velocities versus the numerically differentiated RFB velocities. In the top right corner of each axis, the RMSE between the RFB and KF state estimates is shown.

The TLRL experiment was shown as a full RFB time series in Figure 3, and it is now shown again overlayed with the full KF state estimates in Figure 10. The Figure additionally shows the RMSE of the difference between the two plotted time series, which is the same as that plotted in Figure 8. While there is still some residual motion that cannot be resolved by our filter, the overall fit is quite promising. Appendix A.4 shows the full KF estimate time series of the TLRS and TSRS experiments.

## 4. Discussion and Conclusions

This work introduces and experimentally verifies a structural monitoring solution that incorporates six-component (6C) motion, with the significant advantage of fusing rotational information alongside translation and acceleration. Translation is provided using a GNSS sensor. It is generally assumed that GNSS antennas are not, or are only negligibly, influenced by rotations. However, the rotations that are performed in our experiments, which could occur in the case of slender buildings or be induced by earthquake shaking, will visibly distort the GNSS positions. We show that it is possible to correct the GNSS data with a KF-based fusion scheme and that a significant improvement is achieved, delivering a high potential for 6C structural monitoring.

**Figure A4:**
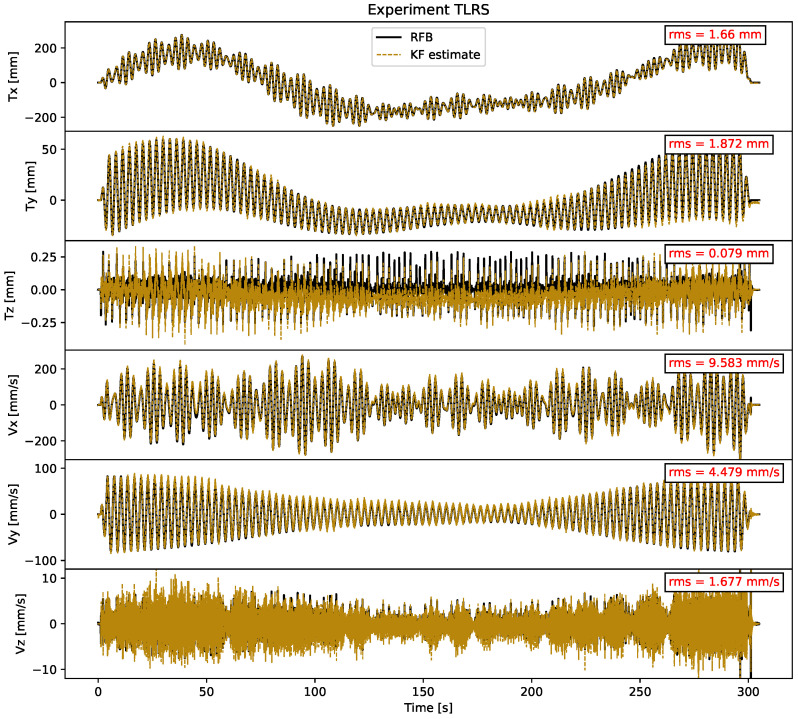
Time series of the full Kalman filter combination for experiment TLRS visualizing KF displacement and velocity estimates and the robot feedback (RFB).

**Figure A5:**
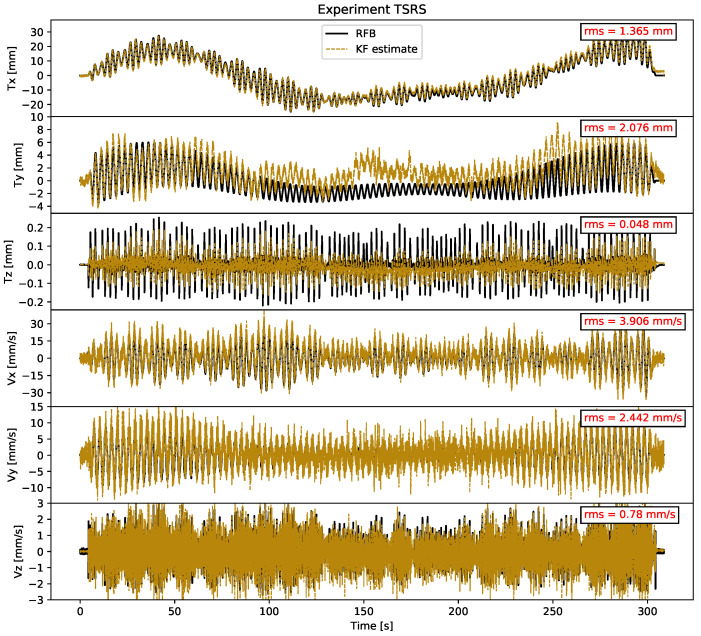
Time series of the full Kalman filter combination for experiment TSRS visualizing KF displacement and velocity estimates and the robot feedback (RFB).

Moreover, in comparison to an accelerometer, GNSS has a limited precision on the order of 2 mm on the horizontal and 5 mm on the vertical axis. Hence, motion amplitudes need to lie above this noise level, in order to be observed by the GNSS receiver. The loss of quality in our TSRS experiment (see Figure A5 in Appendix) in comparison to the TLRS experiment (see Figure A4 in Appendix) is due to the lower signal to noise ratio in the GNSS positions. This becomes evident when the translations become smaller and can no longer be precisely recorded with GNSS. While, in this study, we set the vertical high-noise GNSS positions to zero, forcing the KF to exploit the acceleration data, it is not a generally applicable solution. For applications of a KF to any setting, where the displacement amplitudes fall below the resolution of the GNSS, the equations of this KF would have to be reorganized to more heavily depend on the accelerations, as a more reliable information source, since these are more precise for high-frequency and low-amplitude motion. This type of approach would be required for general seismic monitoring applications, where displacements that are sufficiently large to be recorded by GNSS are only seen in the near field of large earthquakes. Additionally, depending on the application, instruments of very different qualities have to be considered. For general seismic monitoring, where 6C motions from both weak and strong ground shaking need to be recovered, high dynamic range, very broadband and highly sensitive rotational, accelerometric, and GNSS sensors are required. On the other hand, in the case of monitoring known structures, where rotations and accelerations are known to be of larger amplitude, cheaper instruments with lower resolution and dynamic range, such as the IMU used in this study, can be appropriate.

In our simulation of the ambient excitation on a wind turbine, the spectral analysis presented in Figure 4 shows that the rotations evidently distort the acceleration data, and that this effect can be corrected with the incorporation of recorded rotations. After applying the rotation correction, the acceleration reflects the true amplitude of the motion below 10 s period. With the motions simulated in this test, above a 10 s period, the acceleration data are not accurate; for this reason, we depend on GNSS positions, which can observe these longer periods very well. The exploitation of these period-dependent characteristics in our KF formulation yields very broadband results of sufficient precision. This investigation has offered significant insights into the performance of different instruments, mainly because the comparison to the feedback system of the motion generator could be performed. A real quality assessment was possible and, more specifically, it has shown how these measurements are influenced by rotations and how the KF estimation performs during these experiments. The devised KF formulation will allow for us to perform more complex experiments including motions featuring a broader frequency range, i.e., actual data that are obtained from the monitoring of different engineered structures, such as stiffer buildings, or more slender and flexible bridges.

## Figures and Tables

**Table 1:** Relative amplitudes of the three different experiments.

TLRL	TLRS	TSRS
Tx	Tx	Tx/10
Ty	Ty	Ty/10
Rx	Rx/10	Rx/10
Ry	Ry/10	Ry/10

**Table 2:** RFB repeatability. Mean of the RMSE of the differences between the 4 repetitions per experiment.

Experiment	Tx	Ty	Tz	Rx	Ry	Rz
	[mm]	[mm]	[mm]	[mrad]	[mrad]	[mrad]
TLRL	0.1435	0.1053	0.0079	0.0240	0.0792	0.1582
TLRS	4.3604	1.6556	0.0359	0.0636	0.2692	0.3417
TSRS	0.3500	0.1021	0.0188	0.0714	0.1549	0.2835

**Table 3:** The Kalman filter (KF) Algorithm.

Initialization
Set $x_{0}\;\&\;P_{0}$
**Prediction Step**
$x_{k|k-1}=A\;x_{k-1|k-1}+B\;u_{k}$
$P_{k|k-1}=A\;P_{k-1|k-1}\;A^{T}+Q$
**Update Step**
${\mathrm{dy}}_{k}=y_{k}^{GNSS}-C\;x_{k|k-1}-D\;u_{k}$
$K_{k}=P_{k|k-1}\;C\;{\left(C\;P_{k|k-1}\;C^{T}+G\right)}^{-1}$
$x_{k|k}=x_{k|k-1}+K_{k}\;{\mathrm{dy}}_{k}$
$P_{k|k}=\left(I_{6x6}-K_{k}\;C\right)\;P_{k|k-1}$

**Table 4:** Diagonal elements of process noise and measurement noise covariance matrices. The off-diagonal elements are assumed zero, thus making the assumption of uncorrelated noise components.

Experiment	$Q_{1,1}$	$Q_{2,2}$	$Q_{3,3}$	$Q_{4,4}$	$Q_{5,5}$	$Q_{6,6}$	$G_{1,1}$	$G_{2,2}$	$G_{3,3}$
	mm${}^{2}$	mm${}^{2}$	mm${}^{2}$	$\frac{{\mathrm{mm}}^{2}}{s^{2}}$	$\frac{{\mathrm{mm}}^{2}}{s^{2}}$	$\frac{{\mathrm{mm}}^{2}}{s^{2}}$	mm${}^{2}$	mm${}^{2}$	mm${}^{2}$
TLRL/TLRS	$1.25\cdot 10^{-6}$	$1.5\cdot 10^{-7}$	$1\cdot 10^{-13}$	$4\cdot 10^{-6}$	$4\cdot 10^{-7}$	$4\cdot 10^{-12}$	$3\cdot 10^{-8}$	$2\cdot 10^{-8}$	$3\cdot 10^{-10}$
TSRS	$1.25\cdot 10^{-6}$	$1.5\cdot 10^{-6}$	$1\cdot 10^{-13}$	$1\cdot 10^{-4}$	$4\cdot 10^{-5}$	$4\cdot 10^{-12}$	$3\cdot 10^{-8}$	$2\cdot 10^{-8}$	$3\cdot 10^{-10}$

**Figure A2:**
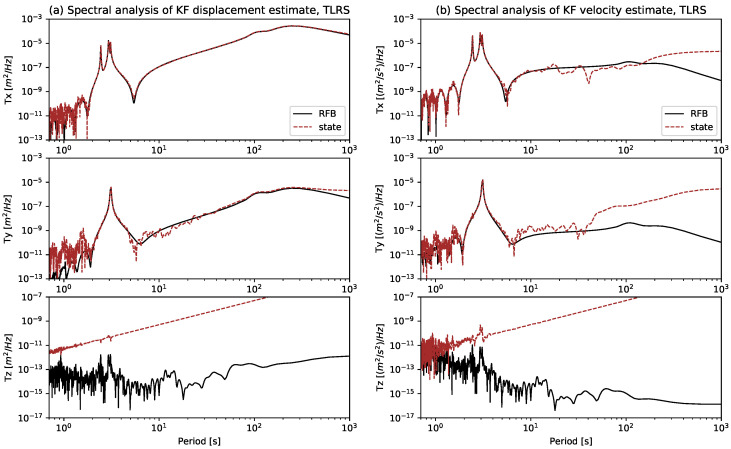
The robot feedback (RFB) is plotted in black and the KF estimate (state) is plotted in brown for the TLRS experiment. The (**a**) shows the KF displacement estimate and (**b**) shows the KF velocity estimate.

**Figure A3:**
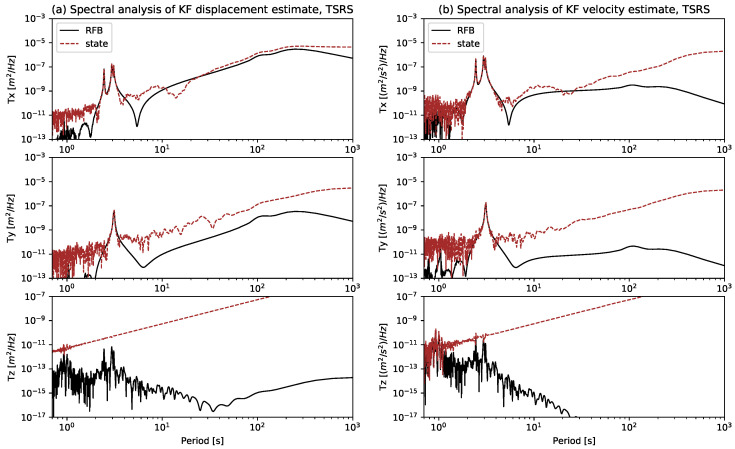
The robot feedback (RFB) is plotted in black and the KF estimate (state) is plotted in brown for the TSRS experiment. The (**a**) shows the KF displacement estimate and (**b**) shows the KF velocity estimate.

## Data Availability

Not applicable.

## Appendix A

### Appendix A.1. Instrument Specifications

**Table A1:** Specification.

Instrument	Weight	Units	Sensitivity	Clip
RFB	60 kg	m and °	$10^{-6}$ m and $10^{-5}$°	1 m and 10–360°
EpiSensor	1.5 kg	m/s/s	$10^{-7}$ m/s/s	4 g
IMU	0.6 kg	°/s	0.8°/h/$\sqrt{\mathrm{Hz}}$	± 490°/s
GNSS	0.5 kg	m	0.004 m	-

### Appendix A.2. Input Motion

Table A2 shows the periods that were used to make up the wind turbine motion performed by the robot. Translation in x or y: Tx, Ty. Rotation around x or y: Rx, Ry. The motion on the x-axis (Tx) is designed to be coupled with the rotation around the y-axis (Ry). The same was done for Ty and Rx. These amplitudes are valid for experiment TLRL. The amplitude adjustments for TLRS and TSRS are visualized in Table 1 in the main part of the article.

**Table A2:** The figure shows the periods that were used to make up the wind turbine motion performed by the robot.

	Tx	Ry		Ty	Rx
Period [s]	Amp. [mm]	Amp. [mrad]	Period [s]	Amp. [mm]	Amp. [mrad]
213.710	145.98	−25.48	207.650	18.19	3.17
543.210	−68.24	11.91	675.610	−7.89	−1.38
132.670	74.98	−13.09	130.090	11.83	2.06
2.999	34.68	−6.05	3.128	20.00	3.49
3.090	32.72	−5.71	3.097	14.87	2.60
2.448	27.17	−4.74	3.063	4.90	0.86
3.022	20.74	−3.62			
2.948	−34.44	6.01			

### Appendix A.3. Time Corrections

One of the most significant difficulties when using different instruments for a combined motion solution, is timing (synchronization). While the GNSS unit and the accelerometer rely on GPS time, which is precise to the 10–20 ns level, the robot and IMU were using computer time, updated by an online NTP server. As a result, the robot timing had to be adjusted with a time drift and shift according to the correlation with the GNSS data. The values can be seen in Figure A1. Additionally, the IMU start time is only precise to a full second and therefore had to be shifted in time as well. Finally, the EpiSensor experiments 7–12 were shifted to the IMU experiments 1–6, using the GNSS recording as a base. The experiment 9 seen in Figure A1 shows a low time drift and was therefore used as the ground truth for all TLRS experiments. Before using the data sets in the Kalman filter, we did a last adjustment; all data sets were interpolated to start at the same time stamp, without changing their sampling rate.

**Table A3:** Instrument information.

Instrument	Time Shift	Time Drift	Sampling Rate
RFB	Yes	Yes	250 Hz
EpiSensor	No	No	250 Hz
IMU	Yes	No	250 Hz
GNSS	No	No	100 Hz

### Appendix A.4. Additional Results

The spectral analysis and the time series of the KF estimates of experiments TLRS and TSRS in comparison to the robot feedback are shown in the following Figures; spectral analysis TLRS see Figure A2a,b, time series TLRS see Figure A4, spectral analysis TSRS see Figure A3a,b and time series TSRS see Figure A5.
